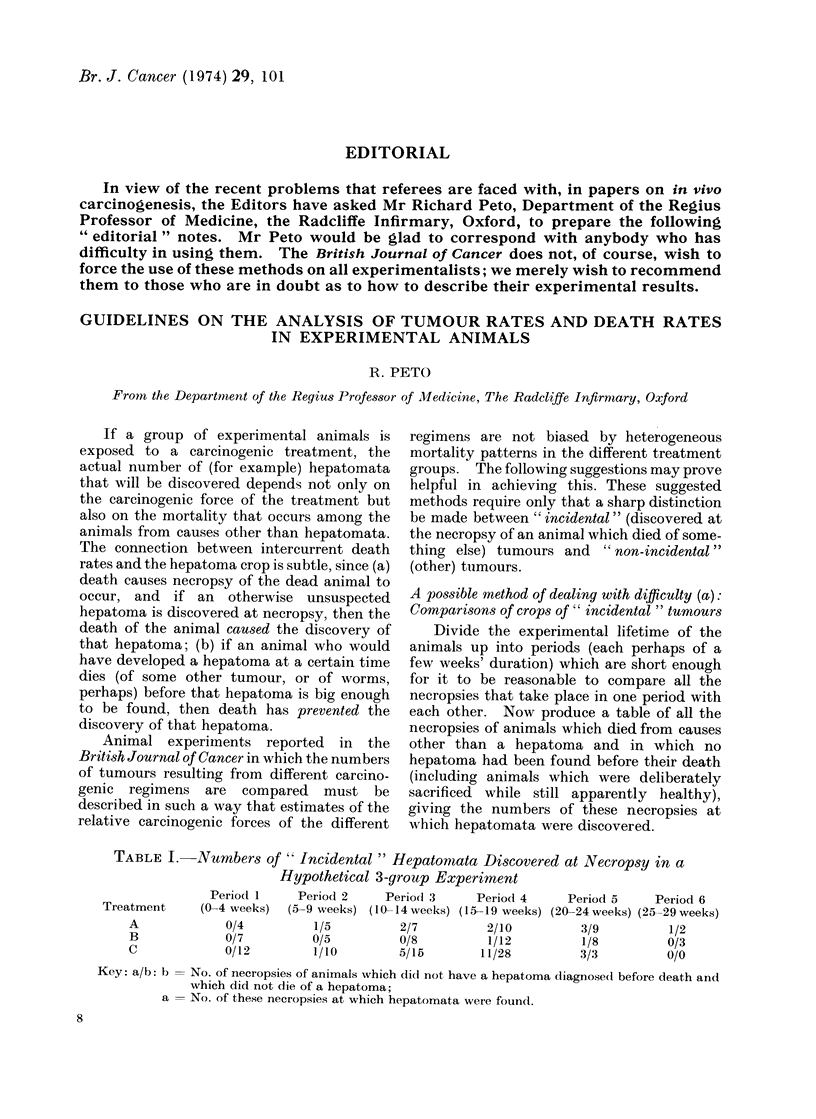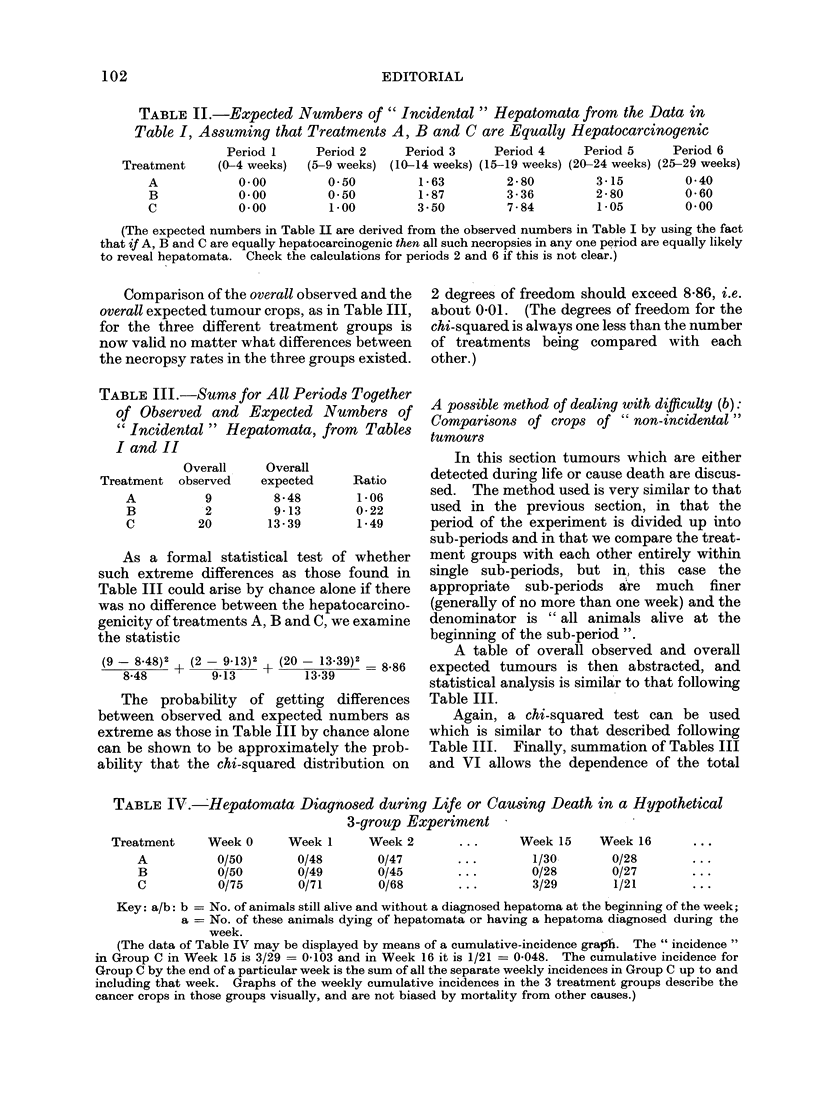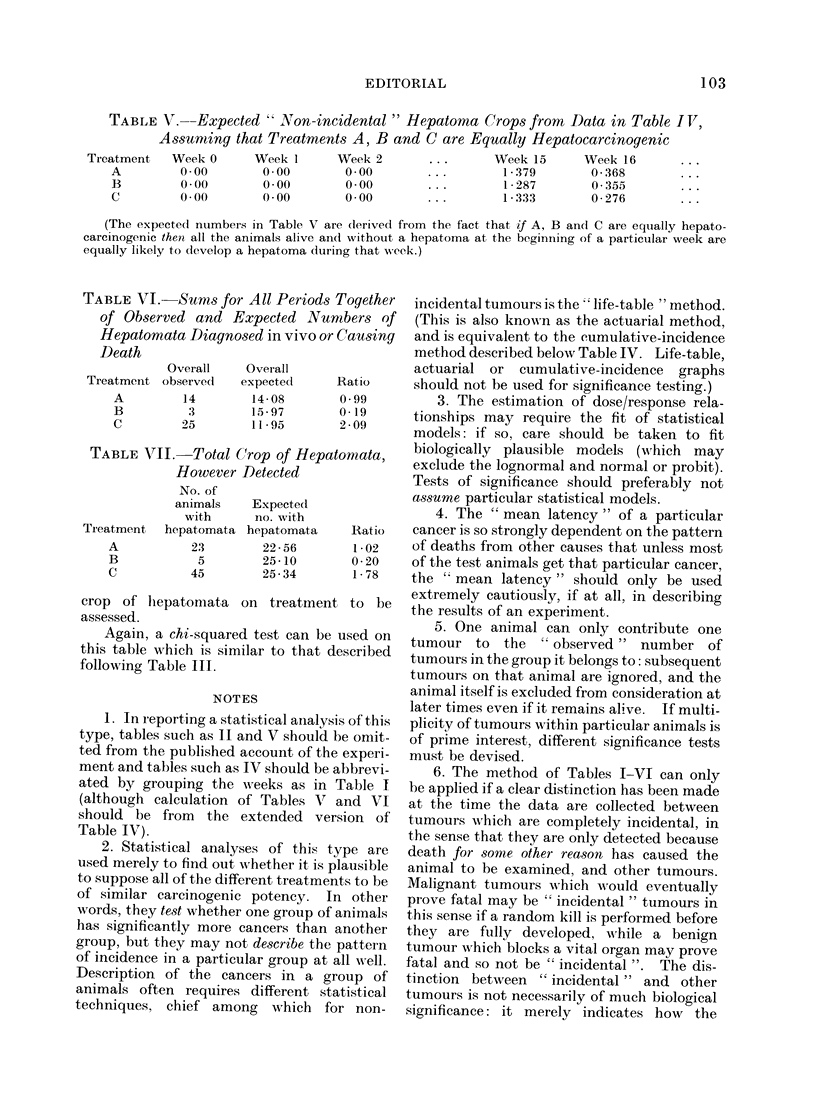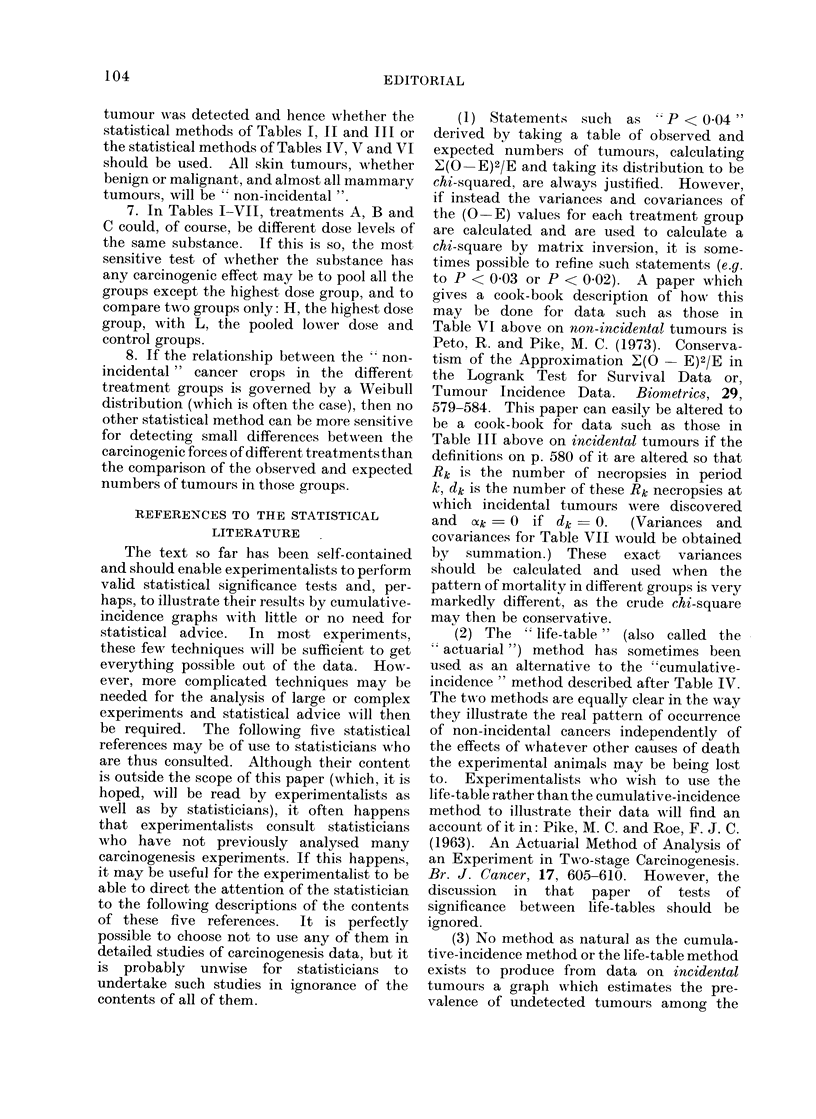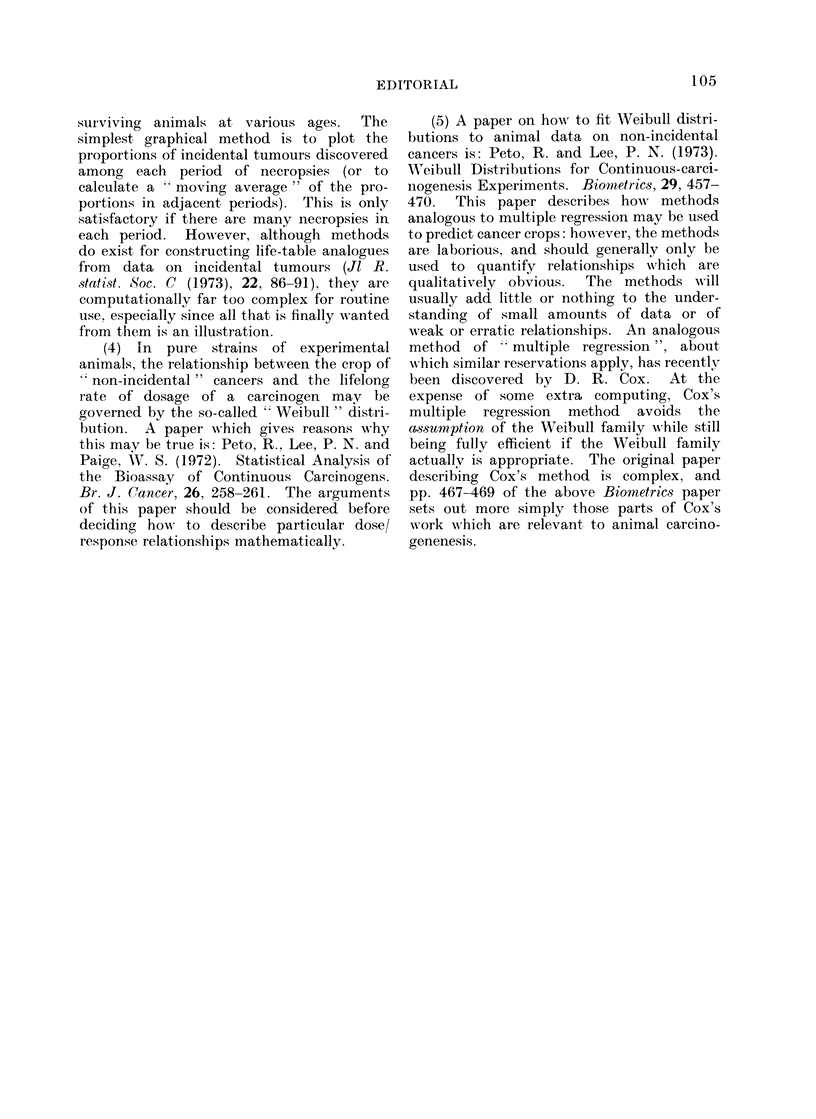# Editorial: Guidelines on the analysis of tumour rates and death rates in experimental animals.

**DOI:** 10.1038/bjc.1974.45

**Published:** 1974-02

**Authors:** R. Peto


*Br. J. Cancer* (1974) **29**, 101

### EDITORIAL

**In view of the recent problems that referees are faced with, in papers on *in vivo* carcinogenesis, the Editors have asked Mr Richard Peto, Department of the Regius Professor of Medicine, the Radcliffe Infirmary, Oxford, to prepare the following " editorial " notes. Mr Peto would be glad to correspond with anybody who has difficulty in using them. The *British Journal of Cancer* does not, of course, wish to force the use of these methods on all experimentalists; we merely wish to recommend them to those who are in doubt as to how to describe their experimental results.**

### GUIDELINES ON THE ANALYSIS OF TUMOUR RATES AND DEATH RATES IN EXPERIMENTAL ANIMALS

R. PETO

*From the Department of the Regius Professor of Medicine, The Radcliffe Infirmary, Oxford*

If a group of experimental animals is exposed to a carcinogenic treatment, the actual number of (for example) hepatomata that will be discovered depends not only on the carcinogenic force of the treatment but also on the mortality that occurs among the animals from causes other than hepatomata. The connection between intercurrent death rates and the hepatoma crop is subtle, since (a) death causes necropsy of the dead animal to occur, and if an otherwise unsuspected hepatoma is discovered at necropsy, then the death of the animal *caused* the discovery of that hepatoma; (b) if an animal who would have developed a hepatoma at a certain time dies (of some other tumour, or of worms, perhaps) before that hepatoma is big enough to be found, then death has *prevented* the discovery of that hepatoma.

Animal experiments reported in the *British Journal of Cancer* in which the numbers of tumours resulting from different carcinogenic regimens are compared must be described in such a way that estimates of the relative carcinogenic forces of the different regimens are not biased by heterogeneous mortality patterns in the different treatment groups. The following suggestions may prove helpful in achieving this. These suggested methods require only that a sharp distinction be made between "*incidental*" (discovered at the necropsy of an animal which died of something else) tumours and "*non-incidental*" (other) tumours.

### *A possible method of dealing with difficulty (a): Comparisons of crops of " incidental " tumours*

Divide the experimental lifetime of the animals up into periods (each perhaps of a few weeks' duration) which are short enough for it to be reasonable to compare all the necropsies that take place in one period with each other. Now produce a table of all the necropsies of animals which died from causes other than a hepatoma and in which no hepatoma had been found before their death (including animals which were deliberately sacrificed while still apparently healthy), giving the numbers of these necropsies at which hepatomata were discovered.

TABLE I.—*Numbers of " Incidental " Hepatomata Discovered at Necropsy in a Hypothetical 3-group Experiment*

| Treatment | Period 1 (0–4 weeks) | Period 2 (5–9 weeks) | Period 3 (10–14 weeks) | Period 4 (15–19 weeks) | Period 5 (20–24 weeks) | Period 6 (25–29 weeks) |
|---|---|---|---|---|---|---|
| A | 0/4 | 1/5 | 2/7 | 2/10 | 3/9 | 1/2 |
| B | 0/7 | 0/5 | 0/8 | 1/12 | 1/8 | 0/3 |
| C | 0/12 | 1/10 | 5/15 | 11/28 | 3/3 | 0/0 |

Key: a/b: b = No. of necropsies of animals which did not have a hepatoma diagnosed before death and which did not die of a hepatoma;
a = No. of these necropsies at which hepatomata were found.

TABLE II.—*Expected Numbers of " Incidental " Hepatomata from the Data in Table I, Assuming that Treatments A, B and C are Equally Hepatocarcinogenic*

| Treatment | Period 1 (0–4 weeks) | Period 2 (5–9 weeks) | Period 3 (10–14 weeks) | Period 4 (15–19 weeks) | Period 5 (20–24 weeks) | Period 6 (25–29 weeks) |
|---|---|---|---|---|---|---|
| A | 0·00 | 0·50 | 1·63 | 2·80 | 3·15 | 0·40 |
| B | 0·00 | 0·50 | 1·87 | 3·36 | 2·80 | 0·60 |
| C | 0·00 | 1·00 | 3·50 | 7·84 | 1·05 | 0·00 |

(The expected numbers in Table II are derived from the observed numbers in Table I by using the fact that *if* A, B and C are equally hepatocarcinogenic *then* all such necropsies in any one period are equally likely to reveal hepatomata. Check the calculations for periods 2 and 6 if this is not clear.)

Comparison of the *overall* observed and the *overall* expected tumour crops, as in Table III, for the three different treatment groups is now valid no matter what differences between the necropsy rates in the three groups existed.

TABLE III.—*Sums for All Periods Together of Observed and Expected Numbers of " Incidental " Hepatomata, from Tables I and II*

| Treatment | Overall observed | Overall expected | Ratio |
|---|---|---|---|
| A | 9 | 8·48 | 1·06 |
| B | 2 | 9·13 | 0·22 |
| C | 20 | 13·39 | 1·49 |

As a formal statistical test of whether such extreme differences as those found in Table III could arise by chance alone if there was no difference between the hepatocarcinogenicity of treatments A, B and C, we examine the statistic

$$\frac{(9 - 8{\cdot}48)^2}{8{\cdot}48} + \frac{(2 - 9{\cdot}13)^2}{9{\cdot}13} + \frac{(20 - 13{\cdot}39)^2}{13{\cdot}39} = 8{\cdot}86$$

The probability of getting differences between observed and expected numbers as extreme as those in Table III by chance alone can be shown to be approximately the probability that the *chi*-squared distribution on 2 degrees of freedom should exceed 8·86, *i.e.* about 0·01. (The degrees of freedom for the *chi*-squared is always one less than the number of treatments being compared with each other.)

*A possible method of dealing with difficulty* (*b*)*: Comparisons of crops of " non-incidental " tumours*

In this section tumours which are either detected during life or cause death are discussed. The method used is very similar to that used in the previous section, in that the period of the experiment is divided up into sub-periods and in that we compare the treatment groups with each other entirely within single sub-periods, but in this case the appropriate sub-periods are much finer (generally of no more than one week) and the denominator is " all animals alive at the beginning of the sub-period ".

A table of overall observed and overall expected tumours is then abstracted, and statistical analysis is similar to that following Table III.

Again, a *chi*-squared test can be used which is similar to that described following Table III. Finally, summation of Tables III and VI allows the dependence of the total

TABLE IV.—*Hepatomata Diagnosed during Life or Causing Death in a Hypothetical 3-group Experiment*

| Treatment | Week 0 | Week 1 | Week 2 | ... | Week 15 | Week 16 | ... |
|---|---|---|---|---|---|---|---|
| A | 0/50 | 0/48 | 0/47 | ... | 1/30 | 0/28 | ... |
| B | 0/50 | 0/49 | 0/45 | ... | 0/28 | 0/27 | ... |
| C | 0/75 | 0/71 | 0/68 | ... | 3/29 | 1/21 | ... |

Key: a/b: b = No. of animals still alive and without a diagnosed hepatoma at the beginning of the week;
a = No. of these animals dying of hepatomata or having a hepatoma diagnosed during the week.

(The data of Table IV may be displayed by means of a cumulative-incidence graph. The " incidence " in Group C in Week 15 is 3/29 = 0·103 and in Week 16 it is 1/21 = 0·048. The cumulative incidence for Group C by the end of a particular week is the sum of all the separate weekly incidences in Group C up to and including that week. Graphs of the weekly cumulative incidences in the 3 treatment groups describe the cancer crops in those groups visually, and are not biased by mortality from other causes.)

TABLE V.—*Expected " Non-incidental " Hepatoma Crops from Data in Table IV, Assuming that Treatments A, B and C are Equally Hepatocarcinogenic*

| Treatment | Week 0 | Week 1 | Week 2 | ... | Week 15 | Week 16 | ... |
|---|---|---|---|---|---|---|---|
| A | 0·00 | 0·00 | 0·00 | ... | 1·379 | 0·368 | ... |
| B | 0·00 | 0·00 | 0·00 | ... | 1·287 | 0·355 | ... |
| C | 0·00 | 0·00 | 0·00 | ... | 1·333 | 0·276 | ... |

(The expected numbers in Table V are derived from the fact that *if* A, B and C are equally hepatocarcinogenic *then* all the animals alive and without a hepatoma at the beginning of a particular week are equally likely to develop a hepatoma during that week.)

TABLE VI.—*Sums for All Periods Together of Observed and Expected Numbers of Hepatomata Diagnosed* in vivo *or Causing Death*

| Treatment | Overall observed | Overall expected | Ratio |
|---|---|---|---|
| A | 14 | 14·08 | 0·99 |
| B | 3 | 15·97 | 0·19 |
| C | 25 | 11·95 | 2·09 |

TABLE VII.—*Total Crop of Hepatomata, However Detected*

| Treatment | No. of animals with hepatomata | Expected no. with hepatomata | Ratio |
|---|---|---|---|
| A | 23 | 22·56 | 1·02 |
| B | 5 | 25·10 | 0·20 |
| C | 45 | 25·34 | 1·78 |

crop of hepatomata on treatment to be assessed.

Again, a *chi*-squared test can be used on this table which is similar to that described following Table III.

NOTES

1. In reporting a statistical analysis of this type, tables such as II and V should be omitted from the published account of the experiment and tables such as IV should be abbreviated by grouping the weeks as in Table I (although calculation of Tables V and VI should be from the extended version of Table IV).

2. Statistical analyses of this type are used merely to find out whether it is plausible to suppose all of the different treatments to be of similar carcinogenic potency. In other words, they *test* whether one group of animals has significantly more cancers than another group, but they may not *describe* the pattern of incidence in a particular group at all well. Description of the cancers in a group of animals often requires different statistical techniques, chief among which for non-incidental tumours is the " life-table " method. (This is also known as the actuarial method, and is equivalent to the cumulative-incidence method described below Table IV. Life-table, actuarial or cumulative-incidence graphs should not be used for significance testing.)

3. The estimation of dose/response relationships may require the fit of statistical models: if so, care should be taken to fit biologically plausible models (which may exclude the lognormal and normal or probit). Tests of significance should preferably not *assume* particular statistical models.

4. The " mean latency " of a particular cancer is so strongly dependent on the pattern of deaths from other causes that unless most of the test animals get that particular cancer, the " mean latency " should only be used extremely cautiously, if at all, in describing the results of an experiment.

5. One animal can only contribute one tumour to the " observed " number of tumours in the group it belongs to: subsequent tumours on that animal are ignored, and the animal itself is excluded from consideration at later times even if it remains alive. If multiplicity of tumours within particular animals is of prime interest, different significance tests must be devised.

6. The method of Tables I–VI can only be applied if a clear distinction has been made at the time the data are collected between tumours which are completely incidental, in the sense that they are only detected because death *for some other reason* has caused the animal to be examined, and other tumours. Malignant tumours which would eventually prove fatal may be " incidental " tumours in this sense if a random kill is performed before they are fully developed, while a benign tumour which blocks a vital organ may prove fatal and so not be " incidental ". The distinction between " incidental " and other tumours is not necessarily of much biological significance: it merely indicates how the tumour was detected and hence whether the statistical methods of Tables I, II and III or the statistical methods of Tables IV, V and VI should be used. All skin tumours, whether benign or malignant, and almost all mammary tumours, will be " non-incidental ".

7. In Tables I–VII, treatments A, B and C could, of course, be different dose levels of the same substance. If this is so, the most sensitive test of whether the substance has any carcinogenic effect may be to pool all the groups except the highest dose group, and to compare two groups only: H, the highest dose group, with L, the pooled lower dose and control groups.

8. If the relationship between the " non-incidental " cancer crops in the different treatment groups is governed by a Weibull distribution (which is often the case), then no other statistical method can be more sensitive for detecting small differences between the carcinogenic forces of different treatments than the comparison of the observed and expected numbers of tumours in those groups.

### REFERENCES TO THE STATISTICAL LITERATURE

The text so far has been self-contained and should enable experimentalists to perform valid statistical significance tests and, perhaps, to illustrate their results by cumulative-incidence graphs with little or no need for statistical advice. In most experiments, these few techniques will be sufficient to get everything possible out of the data. However, more complicated techniques may be needed for the analysis of large or complex experiments and statistical advice will then be required. The following five statistical references may be of use to statisticians who are thus consulted. Although their content is outside the scope of this paper (which, it is hoped, will be read by experimentalists as well as by statisticians), it often happens that experimentalists consult statisticians who have not previously analysed many carcinogenesis experiments. If this happens, it may be useful for the experimentalist to be able to direct the attention of the statistician to the following descriptions of the contents of these five references. It is perfectly possible to choose not to use any of them in detailed studies of carcinogenesis data, but it is probably unwise for statisticians to undertake such studies in ignorance of the contents of all of them.

(1) Statements such as " $P < 0{\cdot}04$ " derived by taking a table of observed and expected numbers of tumours, calculating $\Sigma(O-E)^2/E$ and taking its distribution to be *chi*-squared, are always justified. However, if instead the variances and covariances of the (O−E) values for each treatment group are calculated and are used to calculate a *chi*-square by matrix inversion, it is sometimes possible to refine such statements (*e.g.* to $P < 0{\cdot}03$ or $P < 0{\cdot}02$). A paper which gives a cook-book description of how this may be done for data such as those in Table VI above on *non-incidental* tumours is Peto, R. and Pike, M. C. (1973). Conservatism of the Approximation $\Sigma(O - E)^2/E$ in the Logrank Test for Survival Data or, Tumour Incidence Data. *Biometrics*, **29**, 579–584. This paper can easily be altered to be a cook-book for data such as those in Table III above on *incidental* tumours if the definitions on p. 580 of it are altered so that $R_k$ is the number of necropsies in period $k$, $d_k$ is the number of these $R_k$ necropsies at which incidental tumours were discovered and $\alpha_k = 0$ if $d_k = 0$. (Variances and covariances for Table VII would be obtained by summation.) These exact variances should be calculated and used when the pattern of mortality in different groups is very markedly different, as the crude *chi*-square may then be conservative.

(2) The " life-table " (also called the " actuarial ") method has sometimes been used as an alternative to the "cumulative-incidence " method described after Table IV. The two methods are equally clear in the way they illustrate the real pattern of occurrence of non-incidental cancers independently of the effects of whatever other causes of death the experimental animals may be being lost to. Experimentalists who wish to use the life-table rather than the cumulative-incidence method to illustrate their data will find an account of it in: Pike, M. C. and Roe, F. J. C. (1963). An Actuarial Method of Analysis of an Experiment in Two-stage Carcinogenesis. *Br. J. Cancer*, **17**, 605–610. However, the discussion in that paper of tests of significance between life-tables should be ignored.

(3) No method as natural as the cumulative-incidence method or the life-table method exists to produce from data on *incidental* tumours a graph which estimates the prevalence of undetected tumours among the surviving animals at various ages. The simplest graphical method is to plot the proportions of incidental tumours discovered among each period of necropsies (or to calculate a "moving average" of the proportions in adjacent periods). This is only satisfactory if there are many necropsies in each period. However, although methods do exist for constructing life-table analogues from data on incidental tumours (*Jl R. statist. Soc. C* (1973), **22**, 86–91), they are computationally far too complex for routine use, especially since all that is finally wanted from them is an illustration.

(4) In pure strains of experimental animals, the relationship between the crop of "non-incidental" cancers and the lifelong rate of dosage of a carcinogen may be governed by the so-called "Weibull" distribution. A paper which gives reasons why this may be true is: Peto, R., Lee, P. N. and Paige, W. S. (1972). Statistical Analysis of the Bioassay of Continuous Carcinogens. *Br. J. Cancer*, **26**, 258–261. The arguments of this paper should be considered before deciding how to describe particular dose/response relationships mathematically.

(5) A paper on how to fit Weibull distributions to animal data on non-incidental cancers is: Peto, R. and Lee, P. N. (1973). Weibull Distributions for Continuous-carcinogenesis Experiments. *Biometrics*, **29**, 457–470. This paper describes how methods analogous to multiple regression may be used to predict cancer crops: however, the methods are laborious, and should generally only be used to quantify relationships which are qualitatively obvious. The methods will usually add little or nothing to the understanding of small amounts of data or of weak or erratic relationships. An analogous method of "multiple regression", about which similar reservations apply, has recently been discovered by D. R. Cox. At the expense of some extra computing, Cox's multiple regression method avoids the *assumption* of the Weibull family while still being fully efficient if the Weibull family actually is appropriate. The original paper describing Cox's method is complex, and pp. 467–469 of the above *Biometrics* paper sets out more simply those parts of Cox's work which are relevant to animal carcinogenenesis.